# Effects of anesthetic depth on perioperative T lymphocyte subsets in patients undergoing laparoscopic colorectal cancer surgery: a prospective, parallel-controlled randomized trial

**DOI:** 10.1186/s12871-023-02129-6

**Published:** 2023-05-15

**Authors:** Han Li, Jiachi Li, Conghui Hao, Hengfei Luan, Xiaobao Zhang, Zhibin Zhao

**Affiliations:** 1 Department of Anesthesiology, The Affiliated Lianyungang Hospital of Xuzhou Medical University, No. 6 Zhenhua East Road, Lianyungang, Lianyungang 222000 China; 2 Jinzhou Medical University, No.40 Songpo Road, Jinzhou, Jinzhou 121010 China

**Keywords:** Lymphocyte subsets, Colorectal cancer, Surgical stress, Bispectral index, Anesthetic depth

## Abstract

**Background:**

During the perioperative period, the surgical stress response induced by surgical trauma tends to cause a decrease in peripheral lymphocytes. Anesthetics could reduce the stress response during surgery and prevent sympathetic nerve overexcitation. The goal of this study was to investigate how BIS-guided anesthetic depth affected peripheral T lymphocytes in patients undergoing laparoscopic colorectal cancer surgery.

**Methods:**

A total of 60 patients having elective laparoscopic colorectal cancer surgery were randomly assigned and analyzed (n = 30 for deep general anesthesia, BIS 35, n = 30 for light general anesthesia, BIS 55). Blood samples were collected immediately before anesthesia induction and immediately after operation, 24 h and 5 days postoperatively. The CD4+/CD8 + ratio, T lymphocyte subsets (including CD3 + T cells, CD4 + T cells, and CD8 + T cells), and natural killer (NK) cells were analyzed by flow cytometry. Serum interleukin-6 (IL-6), interferon -ɣ (IFN-ɣ), and vascular endothelial growth factor-α (VEGF-α) were also measured.

**Results:**

The CD4+/CD8 + ratio decreased 24 h after surgery in two groups, but the reduction did not differ between the two groups (*P* > 0.05). The concentration of IL-6 and the numerical rating scale (NRS) score in the BIS 55 group were significantly higher than that in the BIS 35 group 24 h after surgery (*P* = 0.001). There were no intergroup differences in CD3 + T cells, CD4 + T cells, CD8 + T cells, NK cells, VEGF-α, or the IFN-ɣ. Statistical analyses showed no differences between the two groups in the incidence of fever and surgical site infection during hospitalization.

**Conclusions:**

Despite the fact that patients in deep general anesthesia group had low levels of the IL-6 24 h after surgery, the deep general anesthesia was not associated to a positive effect on patients’ peripheral T lymphocytes during colorectal cancer surgery. We found no evidence that peripheral T lymphocyte subsets and natural killer cells were affected by the targeting a BIS of either 55 or 35 in patients undergoing laparoscopic colorectal cancer surgery in this trial.

**Trial registration:**

ChiCTR2200056624 (www.chictr.org.cn).

## Introduction

Worldwide, colorectal cancer (CRC) is the third most commonly diagnosed cancer, and the second for mortality [1, 2]. Surgical removal of CRC is the first-line treatment, postoperative recurrence and metastasis are of great significance for the prognosis of patients [3]. The perioperative period is considered to be a critical window for tumor spread and metastasis, as the impaired immunosurveillance, excessively increased growth factors, and the tumor cells released into circulation caused by surgical procedure [4, 5].

Immunosuppression is commonly observed in patients who underwent trauma and major surgery, which could be characterized by functional disturbances and quantity decline in lymphocytes [6, 7]. T lymphocytes, a major component of adaptive (acquired) immune systems, play important roles in cancer immunity [8]. Several factors can affect the perioperative immune status, and the surgical stress response is a critical factor for perioperative immunosuppression [9]. Therefore, the modulation of the perioperative stress response might be a therapeutic target to preserve the immunological function of patients. Previous studies suggest that anesthetics could counter the surgical stress response and reduce the release of inflammatory cytokines [10, 11]. The bispectral index (BIS) monitor is the most used instrument for instructing anesthetic delivery during operation, and the value of BIS is connected with the anesthetic concentrations and individual sensitivity [12]. However, few studies investigated the influence of BIS-guided anesthetic depth on immunological function.

Therefore, we tested the hypothesis that deep anesthesia (BIS 35) could control surgical stress response effectively, hence CD4+/CD8 + ratio in the deep general anesthesia group is higher than that in the light general anesthesia group (BIS 55). In addition, it was previously reported that high CD4+/CD8 + ratio correlated with stronger cellular immunity [13, 14]. This study aimed to compare the effect of deep anesthesia to light anesthesia on the changes in the CD4+/CD8 + ratio in patients with colorectal resection.

## Methods and materials

### Ethics and registration

#### Ethical approval

for this study (ethics: KY-20211130002-02) was provided by the Ethics Committee of the affiliated Lianyungang Hospital of Xuzhou Medical University, Lianyungang, China on 29 January 2022. The trial was registered in the Chinese Clinical Trial Registry (ChiCTR2200056624) on 09/02/2022 before enrollment. The study protocol followed the CONSORT guidelines. All participants signed written informed consent.

### Patients inclusion and exclusion criteria

We studied patients aged between 18 and 75 years, with an American Society of Anesthesiologists (ASA) physical status of I or II and a BMI range from 18 kg m^− 2^ to 30 kg m^− 2^, who were undergoing elective laparoscopic colorectal cancer surgery under intravenous anesthetic-based general anesthesia without regional anesthesia, clearly understanding and voluntarily participating in the study, and signing the informed consent form. Patients were excluded for the following reasons: use of inhalation anesthesia, epidural anesthesia, or regional anesthesia; conversion from laparoscopic to open surgery; complicated with hematological and immunological diseases; a history of other malignant tumors, radiotherapy, chemotherapy, and blood transfusions within two weeks before the operation, and long-term use of immunosuppressive and anti-inflammatory medications.

### Anesthesia

Patients were routinely monitored by an electrocardiogram (ECG), heart rate (HR), invasive arterial pressure (IBP), pulse oxygen saturation (SpO_2_), central venous pressure (CVP), and BIS once they entered the operating room. Before anesthesia induction, patients were blindly allocated to BIS 35 group or BIS 55 group according to a random number table. All patients were induced by 2.5-3 mg kg^− 1^ propofol, 0.3 ~ 0.4 µg kg^− 1^ sufentanil, and 0.15 mg kg^− 1^ cisatracurium in both two groups. After induction of anesthesia, propofol (4 ~ 12 mg kg^− 1^ h^− 1^) and remifentanil (3 ~ 18 µg kg^− 1^ h^− 1^) were pumped to maintain the anesthesia. Within 10 min of skin incision, the anesthetic depth must be adjusted to the target according to the allocation. It was not permitted to pursue BIS goals at the expense of patient safety.

The BIS ranges from 100 (awake) to 0 (flat line EEG), and as the number decreases, the anesthetic depth increases. During general anesthesia, a BIS value of 40 to 60 was recommended [15]. Based on previous research on anesthetic depth [16, 17] and manufacturer’s recommendations, we chose 35 and 55 as the deep general anesthesia and light general anesthesia group targets, respectively. In the BIS 35 group, patients’ BIS values should be kept between 30 and 40, and in the BIS 55 group, between 50 and 60. Cisatracurium was added in time as needed. A reasonable range (baseline mean atrial pressure ± 30%) of blood pressure fluctuation was established for each patient, and the mean arterial pressure was controlled within this range by pumping nitroglycerin and norepinephrine. At 7 different intraoperative time points (t_0_: preoperative, t_1_: immediately after induction, t_2_: immediately after incision, t_3_: immediately after pneumoperitoneum established, t_4_: 60 min after incision, t_5_: immediately after pneumoperitoneum disappeared, and t_6_: immediately after operation), we measured the MAP, HR, and BIS values for both groups. All drugs administered during the operation were recorded. Non-steroidal anti-inflammatory drugs (NSAIDs) or steroids was not used during the pre-, intra- and postoperative phase. All patients were provided with a patient-controlled intravenous analgesia (PCIA) pump. The PCIA protocol was sufentanil 2 µg kg^− 1^ in 100 ml saline. The parameters were as follows: background dose 2 ml hour^− 1^, PCA 2 ml each time with a locking time of 30 min.

### Blood samples

Flow cytometry was used to determine the number of T lymphocyte subsets (including the CD4+/CD8 + ratio, CD3 + T cells, CD4 + T cells, and CD8 + T cells) and natural killer (NK) cells. Venous blood samples were collected immediately before anesthesia induction (T_0_), immediately after operation (T_1_), and at 24 h and 5 days postoperatively (T_2_ and T_3_). The concentrations of interleukin-6 (IL-6), interferon -ɣ (IFN-ɣ), and vascular endothelial growth factor-α (VEGF-α) were measured by commercial enzyme-linked immunosorbent assay kits (Cloud-clone ELISA kits; USA). A professional laboratory doctor tested the blood sample, and the results were uploaded to an electronic medical record system.

### Clinical measurements

Before leaving the PACU (postanesthesia care unit), intraoperative awareness was evaluated using the Modified Brice Questionnaire. The level of postoperative pain at rest was assessed 1 day after surgery using a numeric rating scale (NRS) ranging from 0 (no pain) to 10 (most pain imaginable). Postoperative 24 h analgesic pump drug residual volume was obtained from the electronic analgesia pump system. Fever and incision site infections that occur during hospitalization are diagnosed by the surgeon in charge of the patient. Fever is defined as a postoperative temperature over 38 °C [18]. The diagnosis of surgical site infection was based upon patient’s combination of clinical and examination feature, laboratory and microbiologic data, and radiography results [19, 20].

### Statistical analysis

On the principle of the intention-to-treat principle, all analyses were performed. All patients who were randomly assigned were included in the intention-to-treat group. The primary outcome was the CD4+/CD8 + ratio 24 h after surgery, while the secondary outcome measures were the number of lymphocyte subsets, intraoperative awareness, emergence time, NRS score, and analgesic pump drug residual volume at 24 h after surgery, as well as the incidences of surgical site infection and fever during hospitalization. According to the published data [21], the mean CD4+/CD8 + ratio at postoperative 24 h was estimated at 1.18 (with a standard deviation [SD] of approximately 0.56) and a difference of 0.5 was considered to be clinically significant. We calculated that 27 patients in each group would be required to detect this difference with a power of 0.90 at a significance level of 0.05 (two-sided). It was intended to enroll 60 patients, with a 10% drop-out rate.

Statistical analysis was performed using the SPSS software (Version 19.0; IBM Corporation, New York). The normality of data distribution was assessed by the Shapiro-Wilk test. Normally distributed continuous data were expressed as the mean ± standard deviation and analyzed using the t-test. The categorical variables were expressed as frequencies (percentages) and analyzed using the chi-square test. The Mann-Whitney U-test was used for continuous variables with a non-normal distribution. The differences in T lymphocyte subsets and NK cells over time were analyzed by Two-way analysis of variance (ANOVA) followed by Bonferroni correction. Statistical significance was set at *P* < 0.05.

## Results

**Fig. 1:**
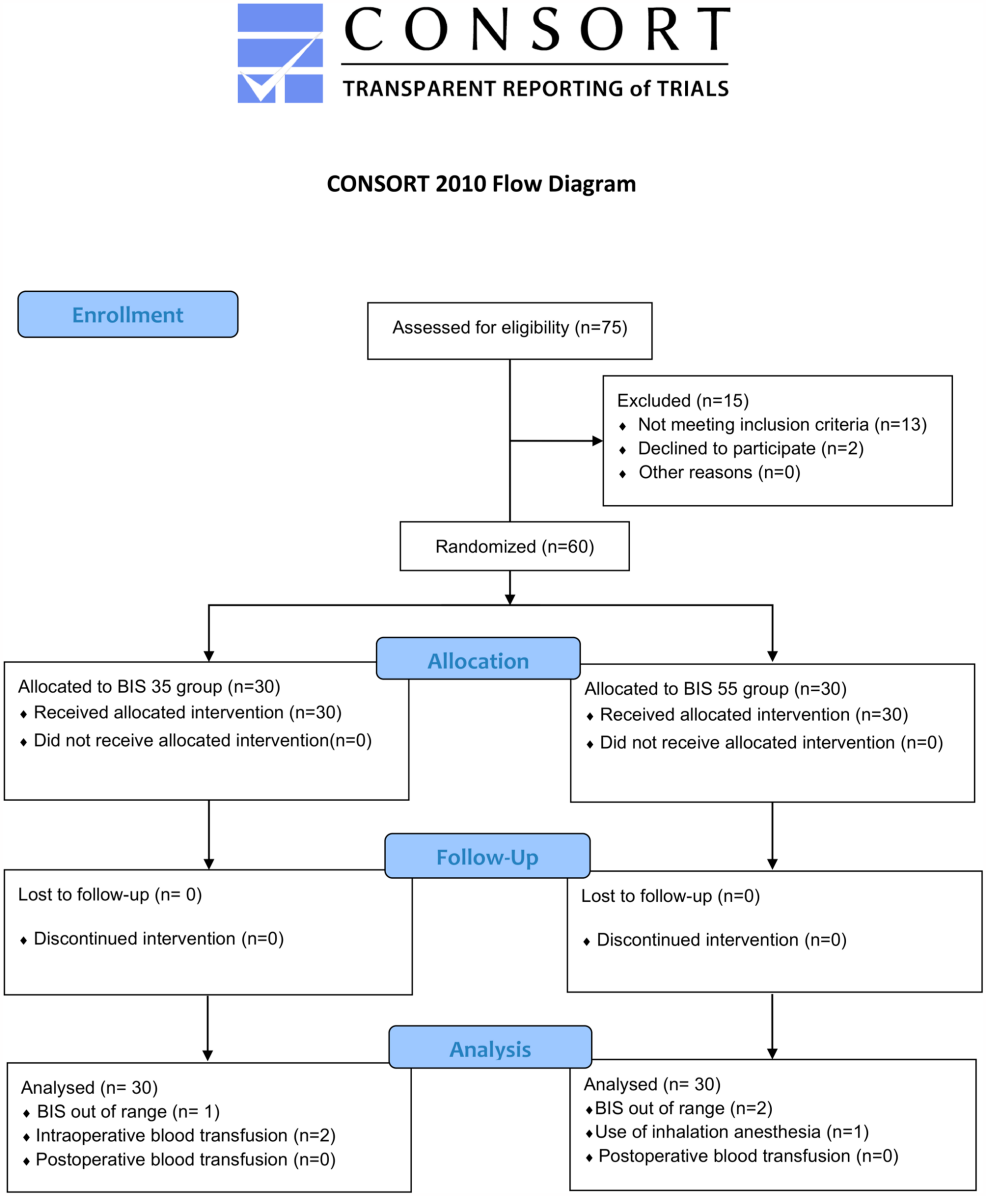
Flow diagram

A total of 75 Patients were enrolled between February 15 and July 31, 2022. Figure 1 depicts the participant flow diagram. After 15 individuals were excluded, 60 patients were randomly divided into two groups and included in the analysis.

Baseline patient characteristics are shown in Table 1. The deep general anesthesia group (BIS 35) and light general anesthesia group (BIS 55) did not differ in age, height, weight, BMI, or sex. No differences were observed between the two groups in the grade of ASA, baseline mean arterial pressure (MAP) or heart rate (HR), operation type, or cancer stage.

**Table 1:** Baseline patient characteristics

	BIS 35 (n = 30)	BIS 55 (n = 30)	*P* Value
Sex			0.184
male	16 (53.3%)	21 (70.0%)	
female	14 (46.7%)	9 (30.0%)	
Age (y)	67 (57 to 69)	66 (58 to 72)	0.544
Heigh (cm)	164 ± 8	166 ± 8	0.495
Weight (kg)	64 ± 10	68 ± 12	0.170
BMI (kg/cm^2^)	24 ± 3	25 ± 4	0.100
ASA			0.592
I	12 (40.0%)	10 (33.3%)	
II	18 (60.0%)	20 (66.7%)	
Baseline MAP (mmHg)	101.5 ± 13.4	97.7 ± 16.1	0.202
Baseline HR (bpm)	72.2 ± 8.6	74.2 ± 8.3	0.663
Type of cancer			0.121
Colon	11 (36.7%)	17(56.7%)	
Rectum	19 (63.3%)	13 (43.3%)	
Stage (I/II/III)			0.842
I	6 (20.0%)	6 (20.0%)	
II	9 (30.0%)	11 (36.7%)	
III	15 (50.0%)	13 (43.3%)	

ASA, American Society of Anesthesiologists; BMI, body mass index; MAP, mean arterial pressure; HR, heart rate. Data are summarised by number (%), median (interquartile range) or mean (standard deviation). *P* < 0.05 was considered statistically different

**Fig. 2:**
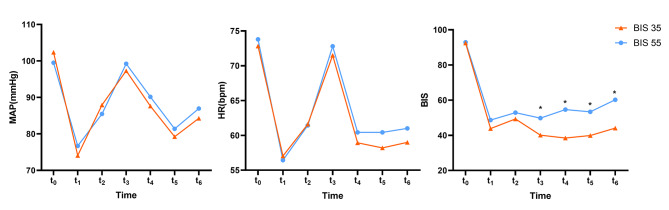
Changes in the MAP, HR and BIS over time in two groups (*P* = 0.501, *P *= 0.798 and *P* < 0.0001, respectively)

Intraoperative characteristics were shown in Table 2. Significant BIS disparity existed between the two groups. The average BIS for the BIS 35 group and the BIS 55 group, respectively, were 40.2 and 53.3 (*P* < 0.001). Figure 2 shows the changes in the MAP, HR and BIS at different time points in two groups. There were no intergroup differences in HR and MAP at 7 different time points. Compared with BIS 35 group, the value of BIS in the BIS 55 group was significantly higher at t_3_, t_4_, t_5_, and t_6_. There were no intergroup differences in duration of anesthesia or surgery, blood loss, infusion or urine volume, norepinephrine or nitroglycerin, or cisatracurium. The doses of total propofol and remifentanil were significantly higher in BIS 35 group (*P* = 0.018, *P* = 0.020 respectively).

**Table 2:** Intraoperative characteristics

		BIS 35 (n = 30)		BIS 55 (n = 30)	*P* Value
Duration of anesthesia (min)		207 (142 to 229)		190 (151 to 227)	0.807
Duration of surgery (min)		178 (132 to 215)		178 (132 to 210)	0.941
BIS		40.2 ± 3.8		53.3 ± 3.2	0.000
MAP (mmHg)		91 (82 to 96)		90 (87 to 94)	0.451
HR (bpm)		60 (54 to 67)		60 (56 to 65)	0.796
Infusion volume (ml)		2178.0 ± 581.0		2298.0 ± 560.0	0.419
Blood loss (ml)		55 (50 to 100)		50 (48 to 105)	0.868
Urine volume (ml)		300 (200 to 420)		210 (150 to 350)	0.131
Colostomy					0.436
Yes		15 (50.0%)		12 (40.0%)	
No		15 (50.0%)		18 (60.0%)	
Total propofol (mg)		1407.0 ± 334.0		1195.0 ± 342.0	0.018
Total propofol dose by body weight (mg/kg)					
		23.0 ± 6.0		18.0 ± 5.0	0.003
Total remifentanil (µg)	1683.0 ± 604.0		1344.0 ± 487.0		0.020
Total remifentanil dose by body weight (µg/kg)					
		27.0 ± 10.0		20.0 ± 8.0	0.006
Total cisatracurium (mg)		21 (16 to 24)		20 (18 to 25)	0.699
Use of norepinephrine					0.297
Yes		19 (63.3%)		15 (50%)	
No		11 (36.7%)		15 (50%)	
Total norepinephrine (µg)		10 (0 to 32)		2 (0 to 12)	0.103
Use of nitroglycerin					0.080
Yes		5 (16.7%)		11 (36.7%)	
No		25 (83.3%)		19 (63.3%)	
Total nitroglycerin (mg)		0 (0 to 0)		0 (0 to 0.27)	0.089

ASA, American Society of Anesthesiologists; BMI, body mass index; MAP, mean arterial pressure; HR, heart rate. Data are summarised by number (%), median (interquartile range) or mean (standard deviation). *P* < 0.05 was considered statistically different

**Fig. 3:**
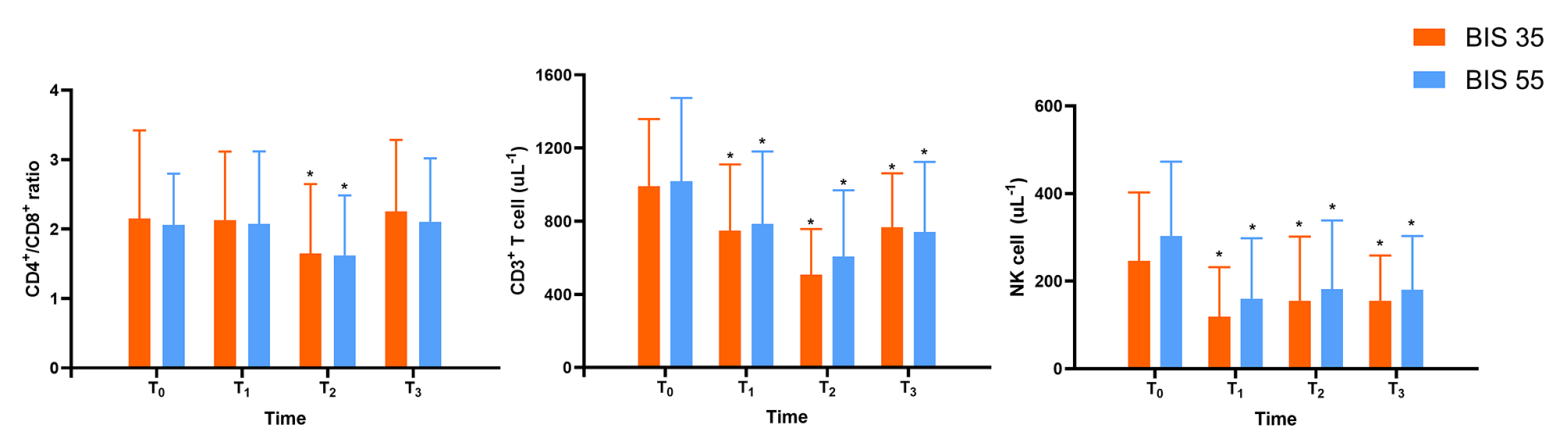
Changes in the CD4+/CD8 + ratio, CD3 + T cell and NK cell were not significantly different in the two groups (*P* = 0.910, *P* = 0.493 and *P* = 0.837, respectively)

Figure 3 shows the changes in CD4+/CD8 + ratio, CD3 + T cells and NK cells over time in two groups. The CD4+/CD8 + ratio decreased at T_1_ in two groups, but the reduction did not differ between the two groups (*P* > 0.05). The CD4+/CD8 + ratio did not differ significantly between the two groups over time (*P* = 0.910). The quantity of CD3 + T cells and NK cells decreased significantly at T_1_, T_2_, and T_3_ compared with T_0_, but the reduction did not differ between the two groups.

**Fig. 4:**
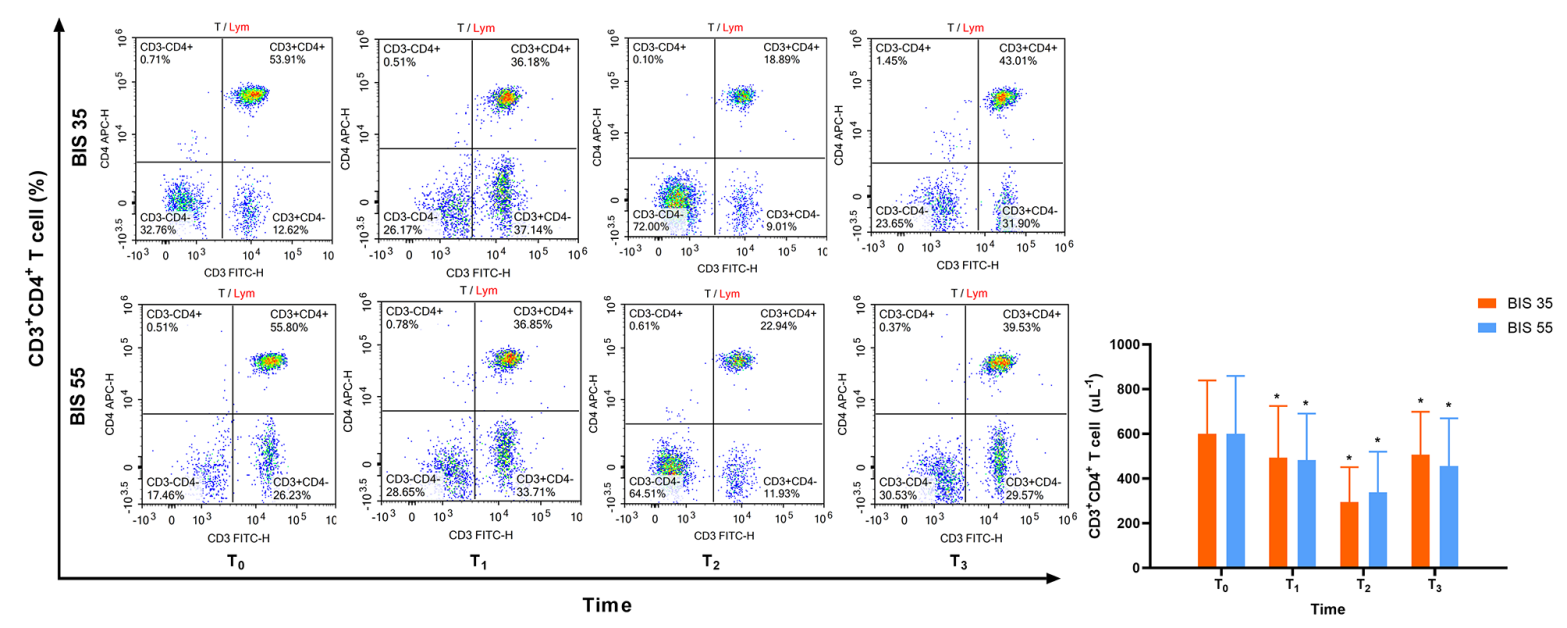
Changes in CD4 + T cell was not significantly different in the two groups (*P* = 0.292)

**Fig. 5:**
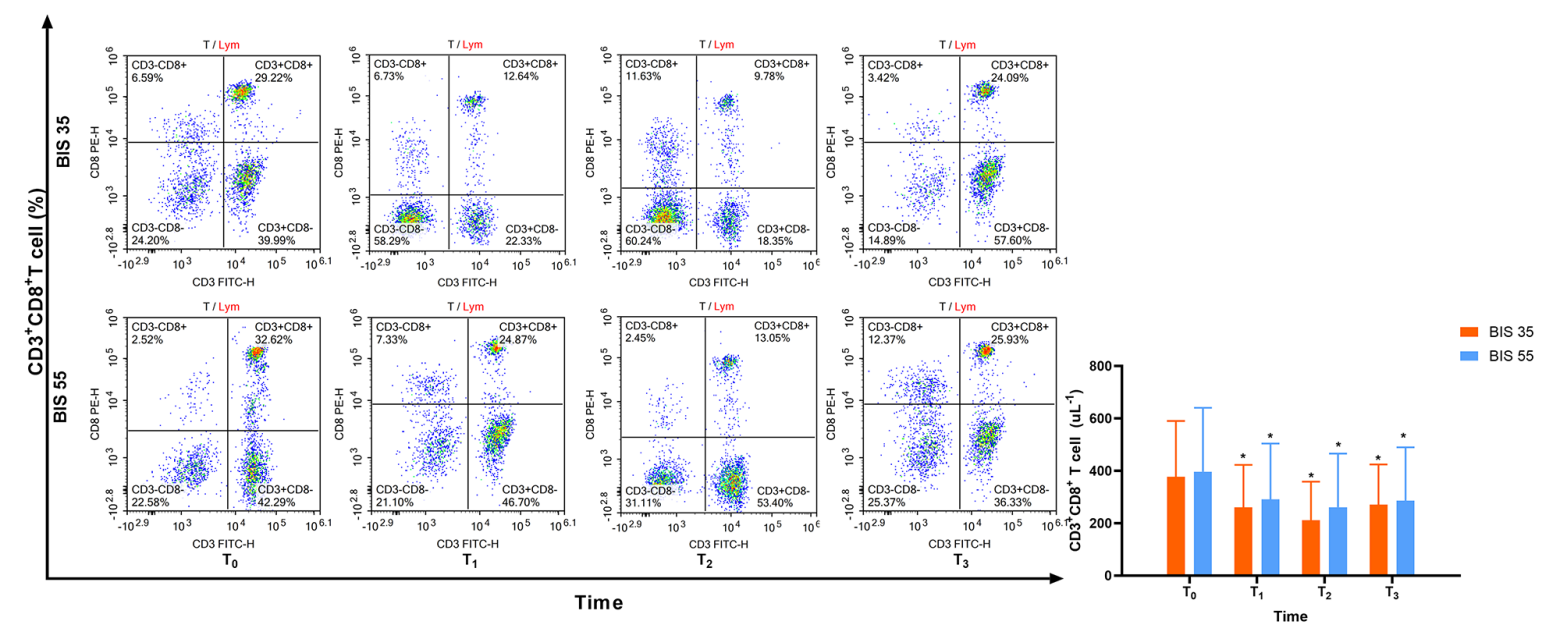
Changes in CD8 + T cell was not significantly different in the two groups (*P* = 0.837)

Figures 4 and 5 depict the changes in CD4 + T cells and CD8 + T cells, respectively. In compared to preoperative levels, the quantity of CD4 + T cells and CD8 + T cells significantly decreased at T_1_, T_2_, and T_3_. There were no significant intergroup differences.

Data on the serum cytokines are shown in Table 3. There were no significant differences in the concentrations of IFN-ɣ and VEGF-α between the two groups. At 24 h after operation, the concentration of IL-6 in the BIS 55 group was noticeably higher than that in the BIS 35 group (*P* = 0.001).

**Table 3:** Perioperative Cytokine Concentrations

	BIS 35 (n = 30)	BIS 55 (n = 30)	Difference (95% CI)	*P* Value
IL-6, pg/ml				
T_0_	2.3 (2.2–2.6)	2.7 (2.2-3.0)	-0.2 (-0.5 to 0.1)	0.181
T_1_	2.5 (2.2-3.0)	2.7(2.2-3.0)	-0.1 (-0.4 to 0.3)	0.745
T_2_	2.6 (2.2-3.0)	3.4 (2.9–3.8)	-0.7 (-1.1 to -0.3)	0.001
T_3_	2.7 (2.2–3.3)	3.0 (2.7–3.7)	-0.4 (-0.8 to 0.1)	0.085
VEGF-α, pg/ml				
T_0_	4.8 (4.0-6.2)	5.7 (4.2–7.8)	-0.7 (-1.9 to 0.3)	0.178
T_1_	5.3 (4.3–7.8)	6.4 (4.7–7.4)	-0.4 (-1.7 to 0.9)	0.511
T_2_	5.4 (4.9–7.3)	6.8 (5.2–8.8)	-1.0 (-2.3 to 0.1)	0.070
T_3_	5.8 (5.4-8.0)	7.0 (5.6–8.5)	-0.9 (-2.2 to 0.1)	0.115
IFN-ɣ, pg/ml				
T_0_	4.9 (4.1–5.8)	5.3 (3.9–7.1)	-0.3 (-1.4 to 0.8)	0.589
T_1_	5.3 (3.6–8.7)	5.0 (3.3–6.9)	0.8 (-0.8 to 2.1)	0.383
T_2_	5.9 (4.0-6.1)	6.1 (4.3–7.9)	-0.9 (-1.9 to 0.3)	0.249
T_3_	5.0 (3.6–6.7)	5.0 (3.7–5.7)	0.3 (-0.7 to 1.4)	0.559

Data are expressed as median (interquartile range) or mean ± SD with difference (95% CI).

T_0_: Preoperative, T_1_: Immediately after operation, T_2_: 24 h after operation, T_3_: 5 days after operation

IL = Interleukin; VEGF = Vascular Endothelial Growth Factor; IFN = Interferon.

Postoperative characteristics is presented in Table 4. The NRS score of the BIS 35 group was significantly lower than the BIS 55 group after 24 h postoperatively (*P* = 0.001). In the two groups, there were no cases of perioperative awareness. There were no intergroup differences in emergence time, analgesic pump drug residual volume at 24 h after surgery, dose of total sufentanil, incidence of fever or surgical site infection during hospitalization.

**Table 4:** Postoperative characteristics

	BIS 35 (n = 30)	BIS 55 (n = 30)	*P* Value
Awareness	0	0	-
Emergence time (min)	12 (10 to 16)	13 (10 to 20)	0.824
POD1 NRS score	2 (1 to 3)	4 (2 to 4)	0.001
Analgesic pump drug residual volume (ml)			0.071
	50.5 (48.1 to 52.0)	49.5 (46.0 to 52.0)	
Total sufentanil (µg)	167.9 ± 22.6	173.3 ± 25.8	0.527
Surgical site infection	9 (20.0)	7 (23.3)	0.559
Fever	8 (26.7)	11 (36.7)	0.405

Data are summarised by number (%), median (interquartile range) or mean (standard deviation). POD1 NRS score, the numerical rating scale score at 1 day postoperatively. Analgesic pump drug residual volume, the analgesic pump drug residual volume at 24 h postoperatively. *P* < 0.05 was considered statistically different

## Discussion

This trial assessed the effect of BIS-guided anesthetic depth on T lymphocytes. The results showed that deep general anesthesia (BIS 35) and light general anesthesia (BIS 55) had similar effects on the changes in CD4+/CD8 + ratio. They also had similar effects on the quantity of CD3 + T cells, CD4 + T cells, CD8 + T cells, and NK cells during laparoscopic colorectal cancer surgery.

The T Lymphocytes are affected by surgical manipulation and anesthetic during operation [22, 23]. The Surgical stress response caused by surgical manipulation is an important reason for the decline of perioperative immune function, which is a complex process of neuroendocrine-metabolic and inflammatory-immune response [9]. A crucial factor in immunosuppression is the inflammatory-immune response, and propofol is known to have an anti-inflammatory effect [24]. Remifentanil and sufentanil are the most commonly used opioids in surgery, with a strong analgesic effect. Furthermore, opioids have been shown to inhibit the hypothalamic-pituitary-adrenal (HPA) and sympathetic adrenal system (SAS) axis overactivation [4, 25, 26]. In their study, Wang et al. [27] discovered that BIS-guided deep anesthesia inhibits the perioperative stress response better than light anesthesia. Quan et al. [28] reported a positive correlation between increasing anesthetic depth and decreased inflammatory response. It was evident that anti-inflammatory treatments are beneficial to cancer patients [29]. Because it is unclear whether actively intervening to regulate anesthetic depth can protect the T lymphocytes and reduce other complications after surgery, therefore, we did the study to compare light general anesthesia and deep general anesthesia in patients undergoing laparoscopic colorectal cancer surgery.

The CD4+/CD8 + ratio is prognostic for cell immunity function and cancer patient outcome. High CD4+/CD8 + ratio has long been thought to be a key indicator of improved prognosis and higher cell immune function, however, there are some controversial opinions in the context of colorectal cancer [13, 14]. Few academics have the opposite opinion to that mentioned above [30]. The CD4+/CD8 + ratio decreased at T_2_ significantly compared with T_0_ in two groups, which was consistent to the results of previous study [21]. In this trial, we found that targeting a BIS of either 55 or 35 had similar effects on the changes in CD4+/CD8 + ratio.

Peripheral mature T lymphocytes are represented by CD3 + T lymphocytes, which subsequently develop into CD4 + T lymphocytes (CD3 + CD4+) and CD8 + T lymphocytes (CD3 + CD8+). By secreting a variety of cytokines and helping CD8 + cytotoxic T cells in dissolving and eliminating tumor cells, CD4 T cells predominantly mediate anti-tumor immunity [31]. Natural killer (NK) cells play an important role in suppressing cancer progression [32]. Surgical stress also inhibited the function of NK cells after colorectal surgery [33]. A previous study suggested that the level of interferon-ɣ in the supernatant indicates NK cell activity [34]. However, there was no significant difference in the levels of IFN-ɣ between the two groups, and deep anesthesia (BIS 35) had no effect on the decline in NK cell activity after laparoscopic colorectal surgery in our study.

It was obvious that patients in two groups showed a significant decline in all types of T lymphocyte cells and NK cells after surgery in this trial. Although the difference between two groups is not statistically significant, patients in the BIS 55 group appear to have higher T lymphocytes and NK cells than that in the BIS 35 group. Several in-vitro and animal studies reported that opioids might trigger immunosuppression and may result in an increase in cancer metastasis [35, 36]. In this trial, patients in the BIS 35 group received more remifentanil than the BIS 55 group, which may be related to the difference in the number of T lymphocytes and NK cells between the two groups. Opioids may theoretically preserve immune function by preventing overactivation of the HPA and SAS axis, but their direct immunosuppressive effects may be more pronounced in clinical background.

Consistent with the result of Quan et al. [28], the concentration of IL-6 in the BIS 55 group was markedly higher than that in the BIS 35 group 24 h after surgery. The NRS score was lower significantly in BIS 35 group, but the postoperative 24 h analgesic pump drug residual volume did not show significant differences between the two groups. The pro-inflammatory cytokine IL-6 is strongly associated with surgical stress response and its intensity [37]. Additionally, IL-6 level always be in line with the intensity of pain. Pain is also a trigger for immunosuppression [38]. Therefore, we considered that the difference in IL-6 level between the two groups caused by the anesthetic depth at 24 h after surgery is the cause of the inconsistency in NRS between groups. Higher IL-6 and NRS levels are both harmful to the immune system. However, the differences of IL-6 and NRS between two groups did not cause a significant intergroup difference in T lymphocytes in this trial. VEGF-α plays a major role in angiogenesis which was important for tumor growth, dissemination, and metastasis [39]. There was no significant difference in serum VEGF-α level between the two groups in this trial. However, these intergroup differences did not affect the T lymphocytes in patients after colorectal surgery.

Norepinephrine and nitroglycerin were used to regulate intraoperative blood pressure in this trial. Sympathetic nerves can influence immune system by activating adrenergic receptors, including negative regulation of T lymphocyte cells maturation and proliferation [40]. Exogenous norepinephrine may affect the number of peripheral T lymphocytes through this pathway. Nitric oxide (NO), a signaling molecule with numerous mechanisms and a multidirectional regulatory effect on the immune system, was released by nitroglycerin after enzymatic hydrolysis [41]. The current study reveals that NO has a facilitative influence on CD4 + T cell differentiation and CD8 + T cell activity [42]. However, the doses of norepinephrine and nitroglycerin were comparable between two groups.

Various factors, including hypothermia, fluid administration, and the patient’s preoperative immunological status, affect the perioperative immune system in addition to anesthetics and surgical stress [43–45]. In the context of a complicated interaction of these factors, the differences between the two groups caused by modulating the anesthetic depth on doses of anesthetics and stress response might be insufficient to affect the patients’ perioperative T lymphocytes.

There were several limitations in this trial. First, we did not achieve our target BIS values in both groups, which might have limited our ability to confirm a difference if one existed. Second, it might be weakly to detect the secondary outcomes due to the small sample size. Third, it was inevitable that the treatments in the ward might affect the results. Last but not least, we only assessed the peripheral T lymphocytes and NK cells in a short time which only partially reflects immune function. Future research is required to observe the patients’ long-term outcomes.

**Conclusion**.

According to our findings, patients who underwent laparoscopic colorectal cancer surgery experienced a prolonged period of lymphopenia. Although there was a tendency toward recovery at 5 days after surgery, it was still relatively low compared to preoperative levels. Despite the fact that patients in deep general anesthesia group had low levels of the pro-inflammatory factor IL-6 24 h after surgery, the deep general anesthesia was not associated to a positive effect on patients’ peripheral T lymphocytes during colorectal cancer surgery. We found no evidence that peripheral T lymphocyte subsets and natural killer cells were affected by the targeting a BIS of either 55 or 35 in patients undergoing laparoscopic colorectal cancer surgery in this trial.

## Data Availability

The datasets used and/or analyzed during the study are available from the corresponding author on reasonable request.

## Abbreviations

BIS: Bispectral index
IL-6: Interleukin-6
IFN-ɣ: Interferon-ɣ
VEGF-α: Vascular endothelial growth factor-α
NK cell: Natural killer cell
CRC: Colorectal cancer
NRS: Numerical rating scale
ECG: Electrocardiogram
IBP: Invasive arterial pressure
MAP: Mean arterial pressure
CVP: Central venous pressure
HR: Heart rate
SpO_2_: Pulse oxygen saturation
ASA: American Society of Anesthesiologists
PCIA: Patient-controlled intravenous analgesia
PACU: Postanesthesia care unit
NASIDs: Non-steroidal anti-inflammatory drugs
ANOVA: Analysis of variance
HPA: Hypothalamic-pituitary-adrenal
SAS: Sympathetic adrenal system
NO: Nitric oxide
